# COVID-19-Associated Pulmonary Aspergillosis in a Series of Complete Autopsies from the Brazilian Amazon

**DOI:** 10.4269/ajtmh.21-1009

**Published:** 2022-01-07

**Authors:** Maria Eduarda Farias, Monique Freire Santana, Luiz Ferreira, Mayla Borba, João Silva-Neto, José Diego Brito-Sousa, Djane Clarys Baía-da-Silva, Guilherme Pivoto João, Fernando Val, Mariana Simão, João Vicente Souza, Felipe Naveca, Gisely Melo, Wuelton Monteiro, Marcus Lacerda

**Affiliations:** ^1^ Instituto de Pesquisa Clínica Carlos Borborema, Fundação de Medicina Tropical Dr. Heitor Vieira Dourado, Manaus, Brazil;; ^2^ Instituto Leônidas & Maria Deane, Fundação Oswaldo Cruz, Manaus, Brazil;; ^3^ Programa de Pós-Graduação em Medicina Tropical, Universidade do Estado do Amazonas, Manaus, Brazil;; ^4^ Departamento de Ensino e Pesquisa, Fundação Centro de Controle de Oncologia do Amazonas, Manaus, Brazil;; ^5^ Departamento de Patologia e Medicina Legal, Universidade Federal do Amazonas, Manaus, Brazil;; ^6^ Instituto de Pesquisas da Amazônia, Manaus, Brazil

## Abstract

Between April and July 2020, and, therefore, prior to the broad recommendation of corticosteroids for severe COVID-19, a total of 50 full autopsies were performed in Manaus. We confirmed two invasive cases of aspergillosis through histopathology and gene sequencing (4%) in our autopsy series. The confirmed invasive aspergillosis incidence seems much lower than expected based on the “probable and possible” definitions, and an individualized approach should be considered for each country scenario. Interestingly, a prolonged length of stay in the intensive care unit was not observed in any of the cases. Timely diagnosis and treatment of fungal infection can reduce mortality rates.

Secondary infections reported in severe acute respiratory disease coronavirus 2(SARS-CoV-2)-infected patients, such as invasive mold disease (IMD), have been well documented in influenza patients, in whom they lead to clinical deterioration and mortality, and require intensive care.^1^ However, there is uncertainty in the literature about whether COVID-19 predisposes to such invasive fungal diseases.^2^

The attempt to estimate the frequency of IMD using the gold standard method (i.e., postmortem examination) showed an IMD worldwide average of 2%, with a predominance of COVID-19-associated pulmonary aspergillosis (CAPA), in a systematic review of autopsies performed in COVID-19 deceased patients.^3^ However, CIs were obviously wide because of the limited number of autopsies performed and varying regional incidence.

That review only included two collated studies from Latin America, with 16 minimally invasive autopsies from southern Brazil, in which no IMD was reported.^4^^,^^5^ In Manaus, one of the first Brazilian capitals to experience the health system collapse triggered by the first wave of COVID-19, in April 2020, a series of complete diagnostic autopsies were performed in a referral unit. Informed consent was signed by legal representatives. The identification of species through molecular methodologies was carried out according to a previously described methodology.^6^

Between April and July 2020, and, therefore, prior to the broad recommendation of corticosteroids for severe COVID-19, 50 complete autopsies were performed (unpublished data). All autopsies presented a clinical picture of severe respiratory distress. In addition, SARS-CoV-2 infection was confirmed in 39 (78%) by reverse transcriptase quantitative real-time polymerase chain reaction, and in 11 (22%) by positive IgM. Of the 50 autopsies, one COVID-19 patient identified with *Aspergillus penicillioides* was reported elsewhere^6^ (case 1). We also report a second case in which *Aspergillus *sp. was identified (case 2). Therefore, we have confirmed two invasive CAPA cases through histopathology and gene sequencing (2 of 50, 4%) in our autopsy series. Clinical characteristics are presented in Table 1. In Figure 1, the histopathology of both patients’ lungs is shown. Both patients also tested positive for galactomannan antigen in the serum. No fungal extrapulmonary lesions were seen. Systematic sampling of macroscopic pulmonary lesions during cleavage was performed. No mucormycosis was diagnosed in this series, although this complication was reported in a patient with COVID-19 from Manaus.^7^ None of the patients used corticosteroids, tocilizumab, or any other immunomodulator, nor antifungal drugs.

**Table 1:** Clinical aspects of COVID-19-associated pulmonary aspergillosis in a series of autopsies

Case no.	Age, y/gender	Comorbidities	Hospitalization, d	ICU, d	MV, d	Imaging exam findings	Sequencing identified (qPCR) lung tissue	GM serum stored blood	Anticoagulant	Attributed cause of death	Radiological characteristics
1	71/male	Systemic arterial hypertension, diabetes mellitus, current smoker, alcoholism, chronic kidney disease	3	3	3	Infiltrate and nodular consolidation in the right lower lobe (chest X-rays)	*Aspergillus penicillioides*	4.29	Yes	Respiratory failure	Ground-glass nodules and subpleural consolidation
2	35/male	Obesity, alcoholism	7	7	7	Bilateral ground-glass infiltrate, bilateral consolidation, pleural effusion (CT scan)	*Aspergillus * spp.	3.62	Yes	Multiple-organ dysfunction syndrome	Bilateral infiltrate with extensive parenchymal opacities

CT = computed tomography; GM = galactomannan; ICU = intensive care unit; MV = mechanical ventilation; qPCR = quantitative polymerase chain reaction.

**Figure 1.**
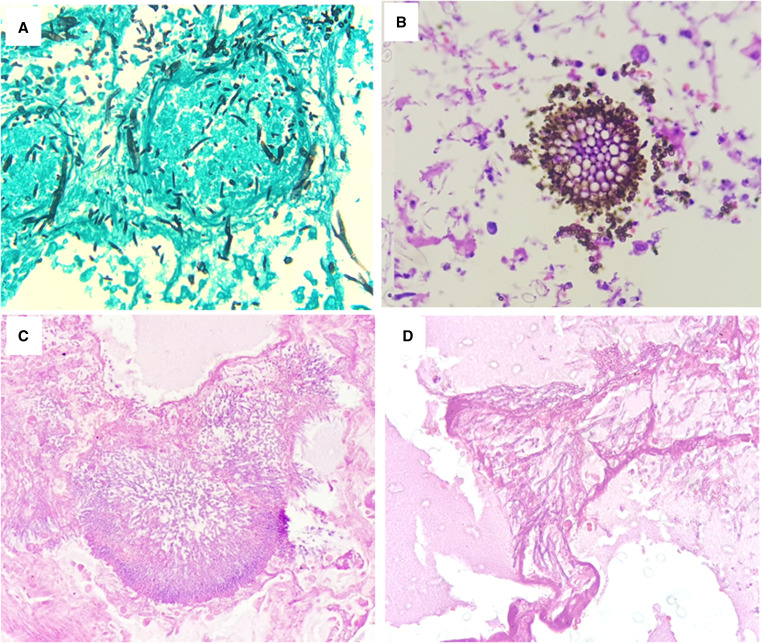
Histopathology of the lungs of the two cases of COVID-19-associated pulmonary aspergillosis. (**A**) Numerous hyphae and fungal spores. (**B**) *Aspergillus* head, allowing visualization of phialides and conidia, with numerous fungal spores. (**C**) Numerous hyphae and fungal spores. Microscopic cavitation surrounded by numerous hyphae and fungal spores. (**D**) Presence of fungal hyphae compatible with *Aspergillus* sp. This figure appears in color at www.ajtmh.org.

In the overall series of 50 autopsied patients, no *Histoplasma capsulatum* was seen, and one patient evolved with candidemia. Of the 50 patients, 43 were hospitalized in the intensive care unit ICU from 1 to 30 days (average ICU stay, 9.8 days). Surprisingly, both patients with CAPA stayed in the ICU only 3 and 7 days.

The difficulty in diagnosing associated pulmonary diseases in COVID-19 patients because of biosafety reasons complicates the management of critically ill patients. This was particularly the case in the first half of 2020, when not much was known about the new SARS-CoV-2 infection, and health systems were collapsing.^8^ Bronchoscopy and serological biomarker-based diagnosis (such as galactomannan), proposed by the European Confederation for Medical Mycology and the International Society for Human and Animal Mycology 2020 consensus,^9^ even under the best biosafety practices, are burdensome and not feasible in most low-income countries, affecting possible diagnoses and leading to the underestimation of CAPA incidence.

On the other hand, even before these guidelines were published, the use of the Clinical Algorithm to Diagnose Invasive Pulmonary Aspergillosis in Critically Ill Patients—the putative diagnosis and extrapolated definitions for influenza-related invasive aspergillosis^10^—could result in overestimating CAPA incidence as a result of the difficulty in distinguishing colonization from infection. Studies have reported worldwide incidences ranging from 8% to 34% in samples from patients under mechanical ventilation.^10^ A meta-analysis reported that CAPA presented with a fatality rate of 54.9% in the ICU.^10^

Brazil, with a vast continental territory, has diverse epidemiological scenarios. The finding of a 4% CAPA incidence in our autopsy series may reflect a better sensitivity of full autopsy analysis, because scattered *Aspergillus*-related lesions may challenge diagnoses when a minimally invasive autopsy is performed. Alternatively, there may be a local higher CAPA incidence in the Amazon compared with southern Brazilian states.

Our finding (4% CAPA incidence) seems to be in the same CI of the meta-analysis published elsewhere (2%).^3^ However, this relatively low incidence should not discourage *Aspergillus* clinical/radiological suspicion in critically ill COVID-19 patients, because coinfection may lead to increased fatality rates. We do not believe that CAPA was a key contributor to the high fatality rates seen in Manaus, which were probably a mere reflex of the lack of preparedness. CAPA incidence confirmed by autopsies seems much lower than expected based on the “probable and possible” definitions, and an individualized approach should be considered for each country scenario. Timely diagnosis and treatment of fungal infection can reduce mortality rates, especially in older patients with underlying diseases. Universal presumptive treatment is still a matter of debate.
